# Synthesis and Properties of MQ Copolymers: Current State of Knowledge

**DOI:** 10.3390/molecules22101768

**Published:** 2017-10-23

**Authors:** Elena Tatarinova, Nataliya Vasilenko, Aziz Muzafarov

**Affiliations:** 1 Enikolopov Institute of Synthetic Polymer Materials of Russian Academy of Sciences Profsoyuznaya st. 70, Moscow 117393, Russia; Tatarinova@ispm.ru (E.T.); n-vasilenko@mail.ru (N.V.); 2 Nesmeyanov Institute of Organoelement Compounds of Russian Academy of Sciences Russia, Vavilova St., 28, GSP-1, V-334, Moscow 119991, Russia

**Keywords:** organosiloxanes, MQ copolymers, MQ resin, polycondensation, hybrid organo-inorganic material

## Abstract

In this review, we discuss currently available studies on the synthesis and properties of MQ copolymers. The data on methods of producing hydrolytic and heterofunctional polycondensation of functional organosilanes as well as the obtaining MQ copolymers based on silicic acids and nature silicates are considered. The ratio of M and Q monomers and the production method determine the structure of MQ copolymers and, accordingly, their physicochemical characteristics. It is shown that the most successful synthetic approach is a polycondensation of organoalkoxysilanes in the medium of anhydrous acetic acid, which reduces the differences in reactivity of M and Q monomers and leads to obtaining a product with uniform composition in all fractions, with full absence of residual alkoxy-groups. The current concept of MQ copolymers is that of organo-inorganic hybrid systems with nanosized crosslinked inorganic regions limited by triorganosilyl groups and containing residual hydroxyl groups. The systems can be considered as a peculiar molecular composites consisting of separate parts that play the role of a polymer matrix, a plasticizer, and a nanosized filler.

## 1. Introduction

MQ copolymers, often called MQ resins, consisting of mono- (M) and tetra- (Q) functional siloxane units that occupy an important place among organosiloxane polymers. Such copolymers have been known for a very long time, the simplicity of their synthesis immediately led to their widespread use as modifying additives in various polymer compositions, so the production of such objects has existed and expanded for more than 50 years. From 1946 to the present, MQ-resins and methods for their production have been patented by various companies: General Electric [1,2,3,4], Dow Corning [5,6,7,8,9], Toray Silicone [10,11], Shin-Etsu Chemical [12], Wacker Chemie AG [13], and many others [14,15,16,17,18,19,20]. The number of patent literature on these objects is very large, and we give only the most significant of them. The peculiarity of these systems is a complete absence of insoluble crosslinked structures even after prolonged high-temperature condensation although their average functionality is usually higher than 2.5, and they have good solubility both in organic solvents and in polydimethylsiloxanes (PDMS). This feature makes it possible to use them widely in compositions with PDMS as reinforcing additives and fillers [21,22], curing catalysts and crosslinking agents [23], additives for production of pressure sensitive adhesives [24], durable coating films, components of sealants and casting compounds, and binding components for production of a new generation of composite materials for various purposes [25,26,27]. It can be argued that MQ-resins are an almost universal modifier for silicone polymers that improves the properties of all formulations. In each case, the role of such additives varies from reinforcement of elastomers to detackification for pressure sensitive adhesives and imparting new properties to release coatings.

The specific structure of the macromolecules—the presence of an inorganic component SiO_4/2_ and an organic component in the R_3_SiO_1/2_ unit—makes it possible to classify these compounds as hybrid nanosized organo-inorganic materials. Аt the present time, the interest in such objects is increasing due to the expanding needs for high quality composite materials. A possibility of introducing various organic alkyl and aryl substituents into the triorganosiloxane unit extends the range of possible structures, including latent functional groups (vinyl, silanol, hydridsilyl), which determine a possibility of further modification.

At present, we can say that the period of empirical selection of reaction conditions for obtaining the necessary technical parameters of MQ systems has come to an end. Investigations of the last decade are mainly aimed at studying the structure of MQ copolymers in relation with their physical characteristics as well as on the study of synthetic approaches that make it possible to obtain the necessary architectural forms of MQ systems purposefully.

**Figure 1:**
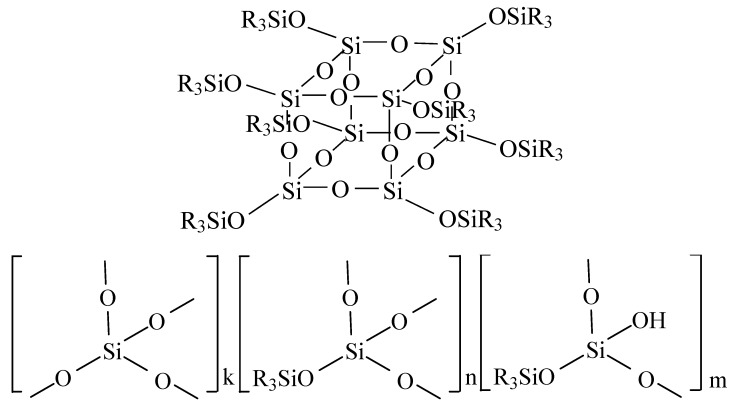
An ideal structure of a cubane M_8_Q_8_ and a real structure of MQ-copolymer.

An ideal structure of MQ copolymer at a ratio of M:Q = 1:1 corresponds to a cubane with M units in the corners, but the actual structure certainly corresponds to more complex and defective structures (Figure 1) [28] with a molecular weight from 1000 to 10,000, which was unambiguously shown in spectroscopic studies and will be considered below.

Methods of obtaining MQ copolymers are very diverse and evolve throughout their lifetimes. First, MQ resins were prepared by a simple cohydrolysis of a mixture of tetra- and monofunctional chloro- or alkoxysilanes [1,5]. Currently, there are three synthetic approaches to the production of such systems: hydrolytic- and heterofunctional polycondensation of organosilicon monomers of M and Q types and trimethylsilylation of silicic acids and natural silicates.

## 2. Results

### 2.1. Obtaining of MQ Copolymers by Hydrolytic Polycondensation

The main problem of a hydrolytic copolycondensation of a mixture of tetra- and monofunctional chloro- [5] or alkoxysilanes [10] is the difference of their hydrolysis rates, which is higher for the tetrafunctional monomer and the formation of homocondensation products of SiX_4_—insoluble compounds corresponding to SiO_4/2_ silicate gels.

As one would expect, this is the most clearly manifested in the cohydrolysis of the most active tetrachlorosilane and trimethylchlorosilane, when the product of cocondensation and a gel of silicic acid and hexamethyldisiloxane are formed. It was necessary to reduce the reactivity of the functional groups and, accordingly, the rate of polycondensation for inhibition of the side reaction of homocondensation. One of the first way for achieving the result was a separate hydrolysis of tetra- and monofunctional chlorosilanes followed by condensation of silanol groups [21]. It is obvious that in this case the silicate component of MQ copolymer will be sufficiently large. Later, a more effective approach was proposed that consisted of the preliminary etherification of a mixture of SiCl_4_ and (CH_3_)_3_SiCl with aliphatic alcohols followed by condensation of corresponding alkoxysilanes [7,29].

Further elaborated technologies of MQ copolymers production mainly used the process of copolycondensation of alkoxy-functional monomers often in the presence of HCl as a catalyst.

The hydrolytic polycondensation method makes it possible to vary the structure and properties of MQ-copolymers by introducing various starting compounds into the process. While using Si(OAlk)_4_ as a Q unit [2] or the product of its partial condensation [12,19,25], a wide range of R_3_SiX triorganosilanes can be used as an M unit. Processes using triorganohaloidsilanes [2,3,14], triorganoalkoxysilanes [2,12], triorganoacetoxysilanes [2], and triorganosilanol [12] have been described where the organic substituents may also have a latent functional nature, for example, contain vinyl or hydridsilyl groups. Later, it was found that hexaorganodisiloxanes [12,19,25], tetramethyldisiloxane [22], and divinyltetramethyldisiloxane [12] might be convenient reagents for the generation of M units.

In the middle of the last century, an approach was proposed that made it possible to synthesize MQ copolymer closest to the ideal structure, that is a silicate cubic with triorganosilyl groups at the angular silicon atoms and consisting in the preliminary synthesis of the monomer of R_3_SiOSiCl_3_ structure. Using this kind of starting compounds, the M and Q units are combined into one molecule, its polycondensation completely prevents the formation of products with a predominance of silicate units, and the polymer has a structure of [R_3_SiOSiO_1.5_]_n_. In this way, MQ structures with phenyl [30] or methyl [31] organic substituents at silicon atom were obtained. In the case of phenyl substituent, the desired products also contained products of partial hydrolysis of the common formula [(C_6_H_5_)_3_SiO]_x_SiCl_4-x_ (x = 1–3), which can be used as precursors for the further production of MQ copolymers. However, in the case of methyl substituent, hydrolysis of the Si-Cl bond and threlease of the trimethylsiloxy-groups and formation of insoluble products close to SiO_4/2_ occurred. Consequently, this approach has not been developed further.

**Scheme 1:**
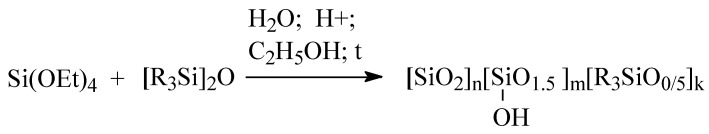
Synthesis of MQ copolymers by hydrolytic copolycondensation.

Currently, the most widely used method of obtaining MQ copolymers is co-condensation of Si(OC_2_H_5_)_4_ or a product of its partial hydrolysis and hexaorganodisiloxane in the presence of HCl and alcohol at temperatures from 0 °C to 90 °C [25,28,32,33,34,35] (Scheme 1).

In this case, the reaction conditions and an M/Q units’ ratio exert a significant influence on such important characteristics as a molecular weight, softening point, content of silanol, and residual alkoxy groups. Varying the M/Q units’ ratio from 1.2 to 0.29 results in a change in the physical state of MQ copolymers from viscous liquids to soluble solid powders. Increasing the boiling time, catalyst concentration, and an amount of Q units lead to an increase in the molecular weight of copolymers. A nature of the acid catalyst affects the ratio of the M and Q units in the copolymer obtained. An increase in the M component content in the initial reaction mixture resulted in a decrease in the content of residual silanol groups in the final product, its molecular weight and thermal stability, and an increase of its hydrophobicity and transparency [36]. So, the simplicity of adjusting the ratio of organic and inorganic units of the structure, leading to obtaining products with such different properties, is a great advantage of the process.

The sol-gel technology of polycondensation production of MQ copolymers has a number of undoubted merits: mild reaction conditions (reactions are carried out at room temperature in the most cases), homogeneity, and high purity of targeted products. These features of the process make it possible to successfully synthesize high quality hybrid nanomaterials with metallic filling (for example, titania) [37,38]. The sol-gel process involving tetra-*n*-butyl titanate made it possible to obtain a high-temperature resistant material with high transparency, where titania crystallization was suppressed by the surrounding MQ copolymer to the size of metal particles of 50 nm [25].

Patent literature devoted to the improvement of MQ copolymers synthesis appears up to the present day, and the composition of MQ copolymers has been considerably expanded., Claimed resins have been made with alkyl, alkenyl, aryl, carboxyl, amide, and alkylamino substituents on the silicon atom in the M unit [39,40,41].

### 2.2. Obtaining of MQ Copolymers by Heterofunctional Polycondensation

**Scheme 2:**
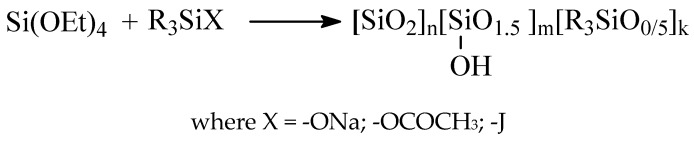
Synthesis of MQ-copolymers by heterofunctional copolycondensation.

Heterofunctional polycondensation of organosilanes can also be a method for obtaining MQ copolymers (Scheme 2).

In early studies, the corresponding tetra(triorganosiloxy)silanes were synthesized by the reaction of sodium triorganosilanolate with silicon tetrachloride [42,43,44], but the yield of the product was low. For example, it was only 18% for tetra(trimethylsiloxy)silane. Later, the authors [45] obtained tetra(trimethylsiloxy)silane by heterofunctional condensation of Si(OC_2_H_5_)_4_ with trimethylacetoxysilane with a yield up to 80%, and a similar result was achieved by the interaction of Si(OC_2_H_5_)_4_ and trimethyliodosilane [46].

A method of synthesis of liquid oligotrimethylsiloxysilanes by heterofunctional polycondensation of trimethylsilyl sulfate with Si(OC_2_H_5_)_4_ or products of its partial condensation in the presence of hexamethyldisiloxane has been patented [47].

The described method of heterofunctional polycondensation of M and Q type monomers was limited by the formation of low-molecular oligomers, and this fact hindered further development and application of this process.

### 2.3. Obtaining of MQ Copolymers Based on Silicic Acids and Silicates

Obtaining MQ copolymers based on cheap natural starting compounds, especially silicates [48,49,50,51,52] and silicic acids and soluble (liquid) glass [53,54,55], which play the role of a tetrafunctional reagent, has been known for a long time and is being developed and used today.

**Scheme 3:**
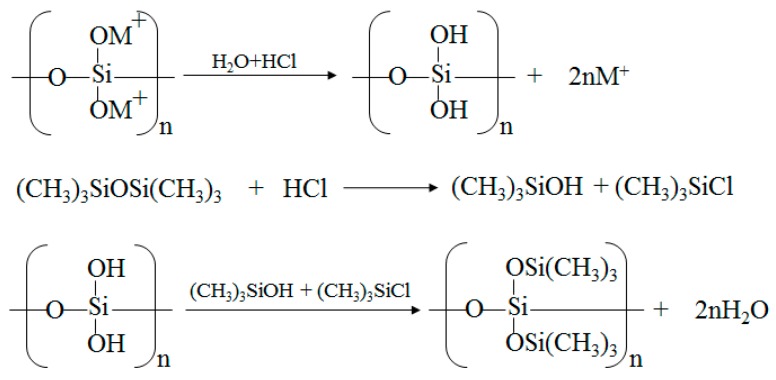
Synthesis of MQ copolymers by trimethylsilylation of natural silicates.

C. W. Lentz [48], the founder of the method of silicate trimethylsilylation, treated various natural silicate materials with an acidic aqueous solution in the presence of isopropyl alcohol and hexamethyldisiloxane (Scheme 3).

The resulting mixture of silicate compounds contained a large amount of low molecular weight products, and the reaction was not considered as a method for producing resins. Later study of trimethylsilylation of natural silicates resulted in obtaining Q_2_M_6_ oligomer with high selectivity [49,51]. In this case such product was formed using different types of silicates—anorthite, labradorite, augite, different in structure and content of various metals. The product had high hydrophobicity and thermal stability up to 435 °C. An undoubted advantage of the method is the fact that cheap natural material or waste of gold and silver extraction serve as a starting reagent, but there are also serious shortcomings. First of all, this is inapplicability of a single developed technology to silicates from various sources, as well as a large consumption of organic solvents, the formation of a significant amount of waste, and, as a result, the formation of low molecular weight MQ copolymers in low yield.

**Scheme 4:**
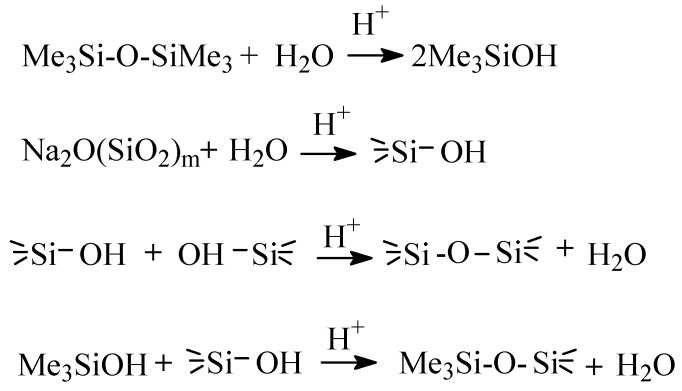
Synthesis of MQ copolymers by trimethylsilylation of liquid glass.

Liquid glass and silicic acids are not natural products, though common, cheap, and large-tonnage ones, and their processing into MQ copolymers is of particular interest to researchers. A currently popular method is the synthesis of MQ copolymers based on a liquid glass [50,51] where sodium silicate (Na_2_SiO_2_)_m_ was used as a starting material, which was treated with chloro- [52] or alkoxytriorganosilane as well as with disiloxanes. In the latter case, the process went on according to the Scheme 4.

A search for optimal conditions for polycondensation of liquid glass with hexamethyldisiloxane—temperature, duration time, reagent feed order, and scaling of the reaction, as well as the selection of a catalyst, resulted in the creation of an efficient technology for obtaining materials, for example, sensitive to pressure [40]. Introduction of phenyl substituents into the tetraorganodisiloxane structure made it possible to obtain MQ systems with significantly improved thermal stability [56], in this case polymers of considerably higher molecular mass were obtained.

### 2.4. Obtaining of MQ-Copolymers by Polycondensation in Active Medium

**Scheme 5:**
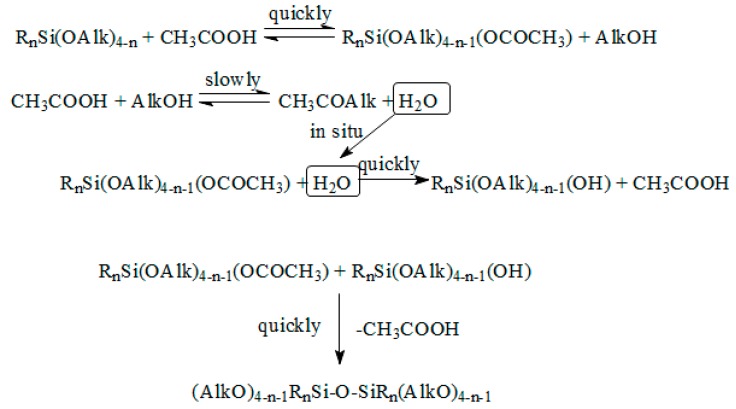
The cascade of reactions of organoalkoxysilane polycondensation in anhydrous acetic acid medium.

Analysis of the available data on the processes of obtaining MQ copolymers allowed us to state that the method of hydrolytic polycondensation is the most universal and in demand. Currently, the most effective way to carry out such a chemical process is a polycondensation of alkoxy-functional silanes of M and Q type in a so-called “active medium” corresponding to anhydrous acetic acid that acts as a reagent and a solvent simultaneously [57]. In this case, water is not introduced into the initial reaction mixture, but the process goes on as a hydrolytic polycondensation due to water release during the reaction. The process is a cascade of interrelated reactions of acidolysis, hydrolysis, and condensation (Scheme 5).

The limiting stage is the interaction of acetic acid and alcohol released during acidolysis of alkoxysilane, with forming without exuding into a separate phase water consumable in situ for hydrolysis of acetoxysilanes.

**Scheme 6:**
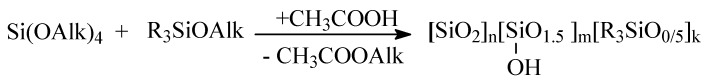
Polycondensation of organoalkoxysilane in anhydrous acetic acid.

The overall scheme of the process looks the classical way (Scheme 6).

Such technique is an effective and universal method of polycondensation ensuring complete homogeneity of the reaction mass throughout the process, at the same time quantitative conversion of alkoxysilyl groups is achieved, differences in the reactivity of the organoalkoxysilanes used are neutralized, which ensures high homogeneity of the resulting product [58]. In order to obtain MQ copolymers, reducing differences in reactivity of monomers M and Q is essential, which leads to obtaining a product with a ratio of M and Q units corresponding completely to the initial amounts introduced and maintained this ratio in all fractions of the copolymer [59].

### 2.5. Structure and Properties of MQ Copolymers

Investigations of the last decade of MQ copolymers are mainly aimed at studying the dependence of their structure and physical characteristics on the reaction conditions. At the end of the last century, physical research methods showed that hydrosilylation-cured MQ copolymers are dense nanoparticles whose cores are represented with Q units and whose surfaces consist of M units [60,61,62].

A series of MQ copolymers—MTQ, MQ, and Q systems, where T is a difunctional unit determining a presence of linear sections—were synthesized to study the effect of the ratio of M and Q unit quantities on the copolymers structure [63]. ESI-FTMS (Bruker, Billerica, MA, USA) mass spectrometry data showed that the structure of the MQ copolymers was the most condensed (cage-like) compared to the more open structure of the Q systems (ladder-like) and the even more mobile structure of the MTQ copolymers. However, in this study, only low-molecular products were investigated.

Nanostructural features of MQ resins were studied by positron annihilation lifetime measurements in terms of the size, numerical concentration, and size distribution of free volumes on samples with different degree of crosslinking [64]. It was shown that the free-volume diameters of the highly crosslinked polysiloxane were found to be and their distributions become broader above T_g_ (150 °C) than those for the lowly crosslinked polysiloxane. Free-volume contents (number density) were lower for the highly crosslinked polysiloxane. The results suggest the heterogeneous structure of MQ resins.

When studying a number of MQ copolymers obtained using the same initial ratio of the M and Q units a significant influence of reaction conditions on the molecular structure of MQ copolymers with identical chemical composition was revealed [65]. All the investigated copolymers were synthesized by hydrolytic polycondensation in an active medium. A different approach consisted first of various initial M units—trimethylmethoxysilane (**MQ1**) or hexamethyldisiloxane in the presence of a catalyst (**MQ2**) were used. Second, pre-synthesized 1,1,1-trimethyl-3,3,3-triethoxydisiloxane (**MQ3**) was condensed by the addition of a catalyst that averaged the product (**MQ4**) structure. In obtaining **MQ5**, the composition of the starting materials was similar to **MQ1**, but the reaction was carried out in stages: condensation of Si(OEt)_4_ was performed, and then (CH_3_)_3_SiOCH_3_ was introduced, thus forming a block variation of MQ copolymer. Polymer **MQ3**, after blocking the residual functional groups with trimethylchlorosilane, corresponded to polymer **MQ6**. All the six MQ copolymers had the same chemical composition M:Q = 1:1, their intrinsic viscosity [η] differed slightly, but significant differences were found in the content of residual hydroxyl groups and glass transition temperatures indicating different mobility of macromolecules, and the structures **MQ2** and **MQ4** obtained in the presence of a catalyst demonstrated the highest mobility. Intrinsic viscosity of all the synthesized MQ copolymers is low, from 0.01 to 0.02 cm^3^/g, in both toluene and THF, that is, all the samples studied have a dense globular structure. However, small differences in [η] of the copolymers in solvents of different nature indicate the ability for conformational changes in the macromolecules depending on the medium. The study of viscosity and relaxation properties showed that all the samples were Newtonian liquids, with practically the same activation energy of frictional flow. Despite a general similarity the rheological parameters of each of the systems differed significantly (Table 1), the shear viscosity of the melts for the least viscous sample **MQ2** was only 100 Pa·s, while the viscosity of the **MQ6** sample is ~10^6^ Pa·s. Each of the MQ copolymers studied has its own relaxation time. They can differ in absolute values by several orders of magnitude. The shortest relaxation time at 90 °C is typical for the **MQ2** sample (~10^−4^ s), and the longest is for the sample **MQ6** (~20 s).

It is important that when studying the results of fractionation of all the copolymers (Table 2), it was shown that the elemental composition of all the fractions for all the variants of MQ copolymers differed very slightly from the original copolymer, M:Q ≈ 1:1 in all cases, but properties of the fractions differed significantly [66].

**Figure 2:**
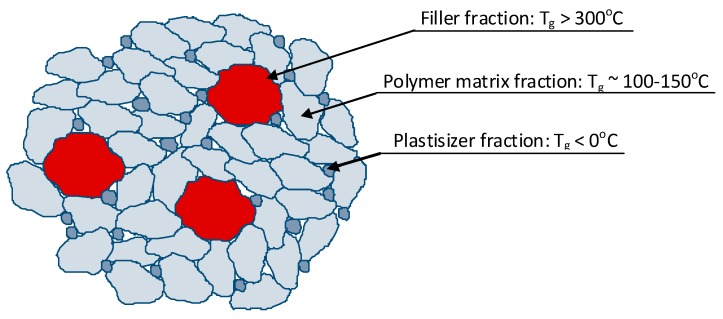
MQ-resins is a molecular composite.

For all MQ copolymers, their high-molecular (Fr. 1) and middle (Fr. 2) fractions were solid substances, and the low-molecular fraction (Fr. 3) was a liquid. Glass transition temperature of high molecular mass fractions was either above the decomposition temperature or above 300 °C, while the average molecular mass of other fractions had glass transition temperature in the range of 80 to 200 °C, and liquid for low molecular mass fractions T_g_ was in the range of −40 °C to −10 °C (Figure 2).

Based on the data obtained, a model describing the MQ-copolymer system as a molecular composite, as a material of complex organization, and of completely uniform composition, where individual fractions are mixed with each other without any restrictions, was proposed. In this system, the most high-molecular formations with a rigid inorganic core play the role of a filler, the middle fractions play the role of a polymer matrix, and the low-molecular copolymers play the role of an effective plasticizer.

A modern study by means of solid-state magic angle spinning (MAS) nuclear magnetic resonance (NMR) spectroscopy of the structure of solid MQ copolymers with common chemical formula [(CH_3_)_3_SiO_0.5_]_m_[SiO_2_]_n_ obtained by polycondensation of alkoxysilanes in active medium showed that MQ copolymers are highly branched polycyclic compounds, or, in other words, tightly crosslinked nanoscale lattices consisting of monofunctional M units = OSi(CH_3_)_3_ and two types of tetrafunctional Q units = (SiO_1/2_)_4_Si and (SiO_1/2_)_3_SiOH. The results of spin–lattice relaxation time T1 measurements of ^29^Si nuclei and analysis of ^29^Si(1H) variable contact time signal intensities yielded quantitative data on relative content of various blocks in the copolymers and showed that MQ copolymers have a dense structure with a core and a shell. Similar results were obtained using of FT-Raman spectroscopy in combination with inelastic neutron scattering [67].

When studying fractions of MQ copolymers in chloroform solutions by pulsed field-gradient NMR spectroscopy, it was found that the diffusion behavior for MQ resin are more characteristic for particle-like macromolecules (for instance, multiarm stars and dendrimers, rather than flexible polymers). However, transition between solutions with different concentrations was not typical for transitions of colloid-like particles [68].

Thus, according to current concepts, MQ copolymers are organo-inorganic hybrid systems with nanosized crosslinked inorganic regions limited by triorganosilyl groups and with a certain number of mobile linear units with residual hydroxyl groups. Such systems might be considered as a peculiar molecular composite consisting of separate parts that play the role of a polymer matrix, a plasticizer, and a nanosized filler [69]. It is clear that the ratio of these parts depends on the reaction conditions and ultimately determines properties of the material. Availability and variability of structure and properties make it possible to consider MQ copolymers as the best example of a commercial hybrid organo-inorganic material of nanometer size [70].

## Figures and Tables

where X = -ONa; -OCOCH_3_; -J

**Table 1:** Main characteristics of MQ copolymers.

Sample, No.	M × 10^−3^ (GPC)	Content of OH-Groups, % Mass	R_hydr_, nm (GPC)	T_g_, °C	η, Pa·s
**MQ1**	3.5	2.26	1.30	55	1.2 × 10^6^
**MQ2**	1.5	0.97	0.80	10	1.0 × 10^2^
**MQ3**	3.5	2.7	1.30	61	1.5 × 10^5^
**MQ4**	1.7	1.4	0.86	34	5.6 × 10^3^
**MQ5**	3.5	2.8	1.30	46	4.7 × 10^4^
**MQ6**	3.5	0	1.30	71	1.4 × 10^6^

**Table 2:** Properties of variants of MQ copolymers.

		Fraction Yield%	MM, GPC	T_g_ °C
**MQ1**	Fr. 1	28	7600	>T_decomp._*
	Fr. 2	44	3900	160
	Fr. 3	28	2000	−26
**MQ2**	Fr. 1	11	5400	319
	Fr. 2	39	2700	78
	Fr. 3	50	1500	38
**MQ3**	Fr. 1	20	5800	>T_decomp._
	Fr. 2	45	3000	110
	Fr. 3	35	1500	−10
**MQ4**	Fr. 1	19	5300	273
	Fr. 2	39	3000	110
	Fr. 3	42	1500	−28
**MQ5**	Fr. 1	21	11,000	>T_decomp._
	Fr. 2	45	3900	180
	Fr. 3	34	1200	−24

* decomposition temperature of the material below the glass transition temperature.
